# Association between rotating night shift work and carotid intima-media thickness among Chinese steelworkers: a cross-sectional survey

**DOI:** 10.5271/sjweh.4031

**Published:** 2022-10-01

**Authors:** Lihua Wang, Shengkui Zhang, Miao Yu, Jianhui Wu, Xiaoming Li, Juxiang Yuan

**Affiliations:** 1 Department of Epidemiology and Health Statistics. School of Public Health. North China University of Science and Technology, Tangshan, Hebei Province, China

**Keywords:** atherosclerosis, cardiovascular disease, China, shift worker

## Abstract

**Objective:**

This study aimed to examine the association between rotating night shift work and subclinical athero­sclerosis among Chinese steelworkers.

**Methods:**

We evaluated 3582 steelworkers who participated in the legally required health examination in 2017. Carotid intima-media thickness (CIMT) was measured using ultrasonography. Different exposure metrics of night shifts collected by face-to-face personal interviews were used to examine the relationship between night shift work and the mean CIMT.

**Results:**

The mean CIMT values were 0.66 (standard deviation 0.22) mm in the study population. Current shift workers shown higher mean CIMT compared to day workers. There were no significant associations between the current shift status, the duration of night shifts, the cumulative number of night shifts, the average frequency of night shifts, and the mean CIMT after all confounding factors adjusted both in male and female.

**Conclusions:**

Rotating night shift work is not associated with subclinical atherosclerosis among steelworkers. Further large-scale prospective longitudinal studies are warranted to confirm our findings.

The timing of 24-hour operations is a major challenge in organizing shift work and one of the major causes of circadian stress (1). The prevalence of shift work was estimated to range roughly 15–20% across industrial countries (2). Shift work may cause the disruption of biological rhythms and perturbation of social and family life, which can negatively influence mental well-being and physiological health (3). Moreover, the International Agency for Research on Cancer (IARC) has classified night shift work as “probably carcinogenic to humans” (Group 2A) in 2019 (4). As it is impossible to avoid night shift work completely, a better understanding of the health effects of shift work will be of great significance for developing preventive strategies for workers.

Cardiovascular diseases (CVD) continue to be the leading cause of death and the largest contributor to premature mortality worldwide (5), causing 40% of deaths in the Chinese population (6). In particular, ischemic heart disease and cerebrovascular disease account for nearly 10 million and 5 million deaths, respectively, globally every year (5). Some studies have shown the association between shift work and clinical cardiovascular outcomes such as peripheral arterial disease, cardiovascular mortality, coronary heart disease (7–11) and atherosclerosis-related vascular events such as myocardial infarction and stroke (12) in both humans and animals (13, 14). In fact, CVD develops over a long period of time, with physical changes beginning decades before the disease manifests itself. However, some of the health hazards of shift work may remain undetected when a disease condition is used as an endpoint, with subsequent difficulty concerning a possible healthy worker effect since workers who cannot tolerate night work may change to day work when the first symptoms of CVD occur (1, 15). Therefore, it is important to include surrogate parameters that describe early subclinical changes (15). Carotid intima-media thickness (CIMT) is a non-invasive measurement obtained by ultrasound. It is a reproducible tool to assess subclinical atherosclerosis (16) and has been shown to reliably predict future CVD events (17, 18) in many kinds of epidemiological studies.

Disrupted or misaligned circadian rhythms promote multiple pathologies, including chronic inflammatory and metabolic diseases such as atherosclerosis (19). However, it has not been determined whether the chronic disruption of circadian rhythms due to night shift work is responsible for carotid atherosclerosis. Apart from that, although demands have been made to improve the quality of exposure assessment of night shift work (20), coarse categorizations are still commonly used to assign exposures in studies of night shift work and subclinical atherosclerosis. The coarse categorizations of night shift work ignore important information that may have an impact on health, such as shift duration and rotation frequency, which may produce measurement error and exposure misclassification within groups. Therefore, it is necessary to study the association between different exposure metrics of night shift work and CIMT examined in the subclinical stage by imaging techniques. Based on the foregoing, in the present study, different exposure metrics, including current shift status, duration of night shifts (years), cumulative number of night shifts (nights) and average frequency of night shifts (nights/month) were used to examine the effects of night shift work on CIMT.

## Methods

### Study design and population

This study was based on cross-sectional data from the occupational population, and was conducted among steelworkers at 11 steel production departments owned by the HBIS Group’s Tangsteel Company in Tangshan City, Hebei Province in North China. All workers at this company underdo a legally required health examination each year. A total of 7661 participants who underwent the annual legally required occupational health examinations were recruited from February to June 2017. There were 4084 workers who volunteered and completed carotid ultrasound examinations. After excluding 205 workers with insufficient shift work data, and 297 workers without complete information on the main covariates of the questionnaire, a total of 3582 participants were included in the final analysis. All participants gave informed consent before taking part in this study. The Ethics Committee of North China University of Science and Technology approved the research in this study (No. 16040).

### Measurement of carotid intima-media thickness

Two trained sonographers performed the assessment of CIMT from both the left and right carotid artery systemusing a high-resolution B-mode topographic ultrasound system using a 7.5 MHz frequency probe (PHILIPS, HD7, China). They were blinded to the research purpose and the study design. Participants were examined in the supine position with their heads rotated in the opposite direction of the probe and with a lateral probe orientation. Common carotid artery (CCA) IMT was measured over a distance of 10 mm proximal to the common carotid bulb on both the left and right sides, excluding focal plaques at the proximal edge, midpoint, or distal edge of the distal CCA in the far wall (21, 22). Three representative measurements were taken per side. Mean CIMT values were calculated from six independent measurements (three per side) (23). To assess intra-reader reproducibility, 5% random workers were re-read with the intra-class correlation coefficients of 0.92.

### Assessment of night shift work

The main work schedule of the present study population was introduced in detail in our previous research (24, 25). In brief, shift work in this study refers to rotating night shifts (the current main four-crew-three-shift system and the historical three-crew-two-shift system). Detailed lifetime employment history was collected in this study by face-to-face personal interviews, and all the reported information was verified with the company’s records. Recruited participants were asked to report whether they were involved in rotating night shift work (working 00:00–6:00 hours) during their employment (current shift status: ‘day work’, ‘ever, ‘current’). If participants responded yes (ever or current), they were asked further about the start and end dates of each shift system, the average number of night shifts worked per month in each shift system. If participants responded no, they were defined as day workers. The duration of night shift work (sum of years spent in all different night shift systems), cumulative number of night shifts (sum of nights spent in all different night shift systems) and average frequency of night shifts (cumulative number of night shifts (nights) divided by cumulative number of months of employment) were derived by using the work schedule information described above.

### Assessment of covariates

The covariates mainly included established risk factors for CVD (26): body mass index (BMI), smoking status, drinking status, diet [dietary approaches to stop hypertension (DASH)], physical activity, sleep duration, insomnia, hypertension, diabetes, dyslipidemia, the use of antihypertensive, antidiabetic and lipid-lowering drugs. Other sociodemographic information was also collected by the questionnaire: age, sex, marital status, educational level. Four mainly related occupational hazard factors including dust, heat stress, noise and carbon monoxide were measured by a qualified third-party company in accordance with the National Occupational Health Standards of the People’s Republic of China (see the supplementary material)

### Statistical analysis

Continuous variables are presented as the means and standard deviations (SD), and between-group comparisons were performed using Student’s t-test or analysis of variance (ANOVA) of normally distributed data. Categorical variables are presented as numbers and percentages, and the chi-square test was used to compare differences among groups. Generalized linear models (GLM) were used to assess the association between different exposure metrics of night shift work (current shift status, duration of night shifts, cumulative number of night shifts, and average frequency of night shifts) and CIMT (continuous variable) using the Statistical Analysis System (SAS) procedure “PROC GENMOD”. Associations between different exposure metrics of night shift work (in quartiles) and CIMT (in quartiles) were reported as odds ratios (OR) and the corresponding 95% confidence intervals (CI) from multiple adjusted logistic regressions. The risk factors and potential confounders were included in the analysis. We fit an unadjusted model and a fully adjusted model including age, sex, marital status, educational level, BMI, smoking status, drinking status, DASH score, physical activity, sleep duration, insomnia, hypertension, diabetes and dyslipidemia. Restricted cubic spline (RCS) models were utilized to visually examine the association between the duration of night shifts (continuous variable), cumulative number of night shifts (continuous variable) and CIMT (continuous variable) with adjustment for potential confounders. Two sensitivity analyses were performed to test the robustness of the results, including further adjustments for the four major occupational hazards and elimination of the last 1% quantile of the duration of night shifts and cumulative number of night shifts. A two-tailed P<0.05 was considered statistically significant. All statistical analyses were performed using SAS V.9.4 (SAS Institute, Cary, NC, USA).

## Results

### General characteristics of the participants

Table 1 shows the basic characteristics of the participants according to the current shift status. The present study included a sample of 3582 participants, with 90.5% being male, a mean age of 46.0 years, and a mean BMI of 25.2 kg/m^2^. The prevalence of hypertension, diabetes and dyslipidemia in the study participants was 32.3%, 13.6%, and 40.1%, respectively. Current smoking, current drinking, and low physical activity were more likely to be reported among current shift workers. Compared with day workers, the sleep duration was relatively shorter among current shift workers. In terms of current health status, current shift workers also showed higher levels of CIMT, BMI, systolic blood pressure, diastolic blood pressure, total cholesterol and LDL-C. As shown in supplementary table S1, compared with female workers, male workers had higher CIMT and BMI levels, and higher proportions of smoking, drinking, hypertension, diabetes, and dyslipidemia. In addition, the CIMT showed age differences (supplementary table S2).

**Table 1:** Basic characteristics of participants according to current shift status. [CIMT=carotid intima-media thickness; DASH=dietary approaches to stop hypertension; BMI=body mass index; HDL-C=high density lipoprotein cholesterol; LDL-C=low density lipoprotein cholesterol; SD= standard deviation].

Variables	Total (N=3582)		Day work (N=710)		Ever (N=754)		Current (N=2118)		P-value ^a^
			
	N (%)	Mean (SD)	N (%)	Mean (SD)	N (%)	Mean (SD)	N (%)	Mean (SD)	
Mean CIMT (mm)		0.66 (0.22)		0.64 (0.21)		0.66 (0.20)		0.67 (0.23)	<0.032
Age (years)		46.0 (7.9)		45.7 (8.9)		46.3 (7.6)		46.0 (7.6)	0.333
Sleep duration (hours)		6.8 (1.2)		7.0 (1.2)		6.8 (1.2)		6.7 (1.2)	<0.001
DASH score		21.6 (2.4)		21.6 (2.4)		21.6 (2.3)		21.6 (2.4)	0.959
Body mass index (kg/m^2^)		25.2 (3.3)		24.9 (3.3)		25.0 (3.2)		25.3 (3.3)	0.003
Systolic blood pressure (mmHg)		129.5 (16.6)		127.6 (16.3)		129.7 (16.2)		130.1 (16.8)	0.003
Diastolic blood pressure (mmHg)		82.8 (10.7)		81.4 (10.5)		83.2 (10.3)		83.1 (10.8)	<0.001
Fasting blood glucose (mmol/L)		6.1 (1.4)		6.1 (1.3)		6.2 (1.5)		6.1 (1.4)	0.175
Total cholesterol (mmol/L)		5.1 (1.0)		5.1 (0.9)		5.1 (1.0)		5.2 (1.0)	0.019
Triglycerides (mmol/L)		1.7 (1.5)		1.6 (1.6)		1.7 (1.6)		1.7 (1.5)	0.698
HDL-C (mmol/L)		1.3 (0.3)		1.3 (0.4)		1.3 (0.3)		1.3 (0.3)	0.232
LDL-C (mmol/L)		3.3 (0.9)		3.2 (0.8)		3.2 (0.9)		3.3 (0.9)	0.047
Sex (male)	3240 (90.5)		620 (87.3)		672 (89.1)		1948 (92.0)		0.001
Age (years)									<0.001
23–29	158 (4.4)		61 (8.6)		27 (3.6)		70 (3.3)		
30–39	594 (16.6)		97 (13.7)		118 (15.7)		379 (17.9)		
40–49	1464 (40.9)		277 (39.0)		311 (41.2)		876 (41.4)		
50–60	1366 (38.1)		275 (38.7)		298 (39.5)		793 (37.4)		
Marital status									<0.001
Unmarried	104 (2.9)		40 (5.6)		17 (2.2)		47 (2.2)		
Married	3382 (94.4)		650 (91.6)		716 (95.0)		2016 (95.2)		
Other	96 (2.7)		20 (2.8)		21 (2.8)		55 (2.6)		
Education level									<0.001
Primary or middle	1049 (29.3)		162 (22.8)		198 (26.3)		689 (32.5)		
High school or college	1900 (53.0)		378 (53.2)		416 (55.2)		1106 (52.2)		
University and above	633 (17.7)		170 (23.9)		140 (18.6)		323 (15.3)		
Smoking status									0.042
Never	1485 (41.5)		315 (44.37)		311 (41.25)		859 (40.56)		
Ever	235 (6.6)		34 (4.49)		63 (8.36)		138 (6.52)		
Current	1862 (52.0)		361 (50.85)		380 (50.40)		1121 (52.93)		
Drinking status									0.003
Never	2091 (58.4)		433 (60.99)		418 (55.44)		1240 (58.55)		
Ever	119 (3.3)		22 (3.10)		30 (3.98)		67 (3.16)		
Current	1372 (38.3)		255 (35.92)		306 (40.58)		811 (38.29)		
Physical activity									<0.001
Low	37 (1.0)		2 (0.28)		6 (0.80)		29 (1.37)		
Moderate	252 (7.0)		40 (5.63)		37 (4.91)		175 (8.26)		
High	3293 (91.9)		668 (94.08)		711 (94.30)		1914 (90.37)		
Sleep duration (h)									<0.001
<6	444 (12.4)		63 (8.9)		79 (10.5)		302 (14.3)		
≥6	3138 (87.6)		647 (91.1)		675 (89.5)		1816 (85.7)		
Insomnia									0.084
No	2324 (64.9)		474 (66.8)		507 (67.2)		1343 (63.4)		
Yes	1258 (35.1)		236 (33.2)		247 (32.8)		775 (36.6)		
Body mass index (kg/m^2^)									0.010
<25	1797 (50.2)		388 (54.7)		394 (52.3)		1015 (47.9)		
25–30	1506 (42.0)		272 (38.3)		312 (41.4)		922 (43.5)		
≥30	279 (7.8)		50 (7.0)		48 (6.4)		181 (8.6)		
Hypertension									0.345
No	2426 (67.7)		492 (69.3)		496 (65.8)		1438 (67.9)		
Yes	1156 (32.3)		218 (30.7)		258 (34.2)		680 (32.1)		
Diabetes									0.345
No	3098 (86.4)		623 (87.8)		642 (85.1)		1833 (86.5)		
Yes	484 (13.6)		87 (12.3)		112 (14.9)		285 (13.5)		
Dyslipidemia									0.034
No	2149 (59.9)		449 (63.2)		466 (61.8)		1234 (58.3)		
Yes	1443 (40.1)		261 (36.8)		288 (38.2)		884 (41.7)		
Antihypertensive	251 (7.0)		53 (7.5)		68 (9.0)		130 (6.1)		0.025
Antidiabetic	80 (2.2)		19 (2.7)		23 (3.1)		38 (1.8)		0.090
Lipid-lowering drugs	40 (1.1)		8 (1.1)		9 (1.2)		23 (1.1)		0.971
Heat stress	1638 (47.2)		244 (35.9)		300 (41.1)		1094 (53.1)		<0.001
Noise	1550 (44.7)		224 (33.0)		228 (31.2)		1098 (53.3)		<0.001
Dust	2230 (64.3)		467 (68.8)		522 (71.5)		1241 (60.2)		<0.001
Carbon monoxide	1511 (43.5)		327 (48.2)		328 (44.9)		856 (41.5)		0.007

a P-values were from Pearson’s χ2 test for categorical variables and analysis of variance (ANOVA) for continuous variables.

### Association between duration of night shifts and CIMT

The CIMT values for the whole participants were 0.66 (SD 0.22) mm (table 1). The GLM analysis revealed positive and significant associations of different exposure metrics of night shift work with the CIMT in the unadjusted model (table 2). After further adjusting all other confounding factors (age, sex, marital status, educational level, BMI, smoking status, drinking status, DASH score, physical activity, sleep duration, insomnia, hypertension, diabetes and dyslipidemia), the current shift status, duration of night shifts, cumulative number of night shifts and average frequency of night shifts did not show significant associations with the CIMT (table 2).

**Table 2:** Associations of different exposure metrics of night shift work with mean carotid intima-media thickness (CIMT) from generalized linear models. [CI=confidence interval; BMI=body mass index; DASH=dietary approaches to stop hypertension.]

Exposure metrics	N (%)	Unadjusted			Adjusted ^a^		
	
		β	95% CI	P-value	β ^a^	95% CI ^a^	P-value ^a^
Current shift status							
Day work	710 (19.8)	0 (ref)			0 (ref)		
Ever	754 (21.1)	0.023	0.001–0.045	0.044	0.014	-0.006–0.034	0.173
Current	2118 (59.1)	0.024	0.006–0.042	0.010	0.015	-0.002–0.032	0.076
P for trend				0.019			0.102
Duration of night shifts (years)							
Day work	710 (19.8)	0 (ref)			0 (ref)		
Q1 (1–12)	721 (20.1)	-0.033	-0.053– -0.011	0.003	0.013	-0.008–0.034	0.203
Q2 (13–20)	697 (19.5)	-0.009	−0.030–0.013	0.403	0.013	-0.008–0.034	0.222
Q3 (21–28)	707 (19.7)	0.039	0.017–0.062	<0.001	0.015	-0.006–0.036	0.151
Q4 (29–43)	747 (20.9)	0.096	0.077–0.125	<0.001	0.018	-0.003–0.040	0.096
P for trend				<0.001			0.107
Cumulative number of night shifts (nights)							
Day work	710 (19.8)	0 (ref)			0 (ref)		
Q1 (43–1209)	717 (20.0)	-0.035	-0.054– -0.014	0.002	0.012	-0.009–0.033	0.247
Q2 (1210–2068)	718 (20.0)	-0.015	−0.036–0.006	0.177	0.009	-0.012–0.030	0.405
Q3 (2069–2684)	719 (20.1)	0.054	0.033–0.078	<0.001	0.024	0.003–0.046	0.023
Q4 (2685–5239)	718 (20.0)	0.091	0.072–0.119	<0.001	0.015	-0.006–0.037	0.167
P for trend				<0.001			0.084
Average frequency of night shifts (nights/month)							
Day work (nights/month)	710 (19.8)	0 (ref)			0 (ref)		
≤3	740 (20.7)	0.028	0.006–0.051	0.015	0.006	-0.014–0.026	0.526
4–7	1807 (50.4)	0.015	-0.004–0.033	0.127	0.019	0.002–0.037	0.029
>7	325 (9.1)	0.066	0.039–0.098	<0.001	0.011	-0.014–0.037	0.401
P for trend				0.002			0.054

a Adjusted for age, sex, marital status, educational level, BMI, smoking status, drinking status, DASH score, physical activity, sleep duration, insomnia, hypertension, diabetes, dyslipidemia.

**Figure 1:**
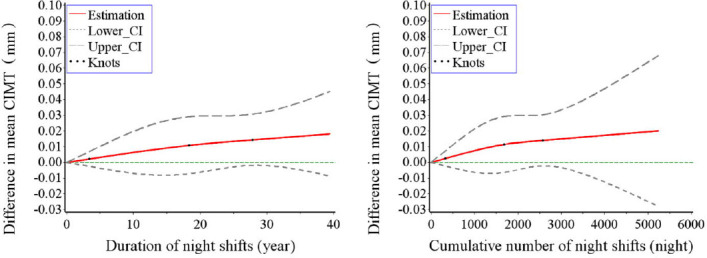
Associations of different continuous exposure metrics of night shift work with mean carotid intima-media thickness (CIMT) (continuous variable) from restricted cubic spline models. “Difference in mean CIMT” represents the difference in mean CIMT (mm), where the reference values for the duration of night shifts and the cumulative number of night shifts are all 0 (day work). Adjusted for age, sex, marital status, educational level, body mass index (BMI), smoking status, drinking status, dietary approaches to stop hypertension (DASH) score, physical activity, sleep duration, insomnia, hypertension, diabetes, dyslipidemia.

Table 3 shows the results from the logistic regression model, which was performed to maximize the difference between the outcomes of exposure (Q4 versus Q1 quartile of the CIMT distribution). When the outcome was dichotomized as CIMT in the Q4/Q3/Q2 quartile versus Q1 quartile of the CIMT distribution, there were no significant associations between different exposure metrics of night shift work and the CIMT after all confounding factors adjusted (table 3). No significant associations were observed among male or female workers (supplementary table S4). Moreover, positive associations (without statistical significance) were observed between the duration of night shifts, cumulative number of night shifts and the CIMT in the RCS models (figure 1).

**Table 3:** Multivariate adjusted odds ratio (OR) ^a^ between quartile of the mean carotid intima-media thickness (CIMT) and different exposure metrics of night shift work. [CI=confidence interval; BMI=body mass index; DASH=dietary approaches to stop hypertension].

Exposure metrics	N (%)	OR (95% CI)		

		Q2 versus Q1	Q3 versus Q1	Q4 versus Q1
Current shift status				
Day work	710 (19.8)	1.00	1.00	1.00
Ever	754 (21.1)	0.97 (0.70–1.33)	1.27 (0.95–1.69)	1.32 (0.95–1.83)
Current	2118 (59.1)	0.86 (0.67–1.15)	0.63 (0.50–0.81)	0.91 (0.96–1.20)
P for trend		0.210	<0.001	0.165
Duration of night shifts (years)				
Day work	710 (19.8)	1.00	1.00	1.00
Q1 (1–12)	721 (20.1)	0.89 (0.65–1.20)	0.65 (0.49–0.86)	1.06 (0.75–1.49)
Q2 (13–20)	697 (19.5)	0.95 (0.70–1.30)	0.64 (0.48–0.87)	1.02 (0.73–1.44)
Q3 (21–28)	707 (19.7)	0.76 (0.55–1.05)	0.78 (0.58–1.05)	0.91 (0.65–1.27)
Q4 (29–43)	747 (20.9)	0.93 (0.65–1.33)	1.22 (0.89–1.67)	0.97 (0.68–1.37)
P for trend		0.395	0.219	0.482
Cumulative number of night shifts (nights)				
Day work	710 (19.8)	1.00	1.00	1.00
Q1 (43–1209)	717 (20.0)	0.87 (0.64–1.19)	0.63 (0.48–0.84)	1.04 (0.74–1.47)
Q2 (1210–2068)	718 (20.0)	0.89 (0.66–1.21)	0.61 (0.45–0.82)	0.91 (0.65–1.27)
Q3 (2069–2684)	719 (20.1)	0.86 (0.62–1.20)	0.83 (0.61–1.13)	1.09 (0.78–1.52)
Q4 (2685–5239)	718 (20.0)	0.90 (0.63–1.28)	1.26 (0.92–1.73)	0.93 (0.66–1.31)
P for trend		0.475	0.095	0.636
Average frequency of night shifts (nights/month)				
Day work (nights/month)	710 (19.8)	1.00	1.00	1.00
≤3	740 (20.7)	0.98 (0.71–1.35)	1.26 (0.94–1.69)	1.23 (0.89–1.72)
4–7	1807 (50.4)	0.92 (0.71–1.19)	0.60 (0.47–0.77)	0.96 (0.73–1.28)
>7	325 (9.1)	0.57 (0.37–0.87)	0.93 (0.64–1.36)	0.80 (0.54–1.19)
P for trend		0.049	<0.000	0.160

a Adjusted for age, sex, marital status, educational level, BMI, smoking status, drinking status, DASH score, physical activity, sleep duration, insomnia, hypertension, diabetes, dyslipidemia.

To avoid the influence of the maximum value on the fitting result of the RCS models, we removed the last 1% quantile of the duration of night shifts and cumulative number of night shifts, and the results remained stable (supplementary figure S1). Considering that dust, heat stress, noise, and carbon monoxide are the main occupational hazards for current steelworkers, we further adjusted these exposures on the basis of Model 3, as shown in table 3, and the results remained stable (supplementary table S4).

## Discussion

In this cross-sectional study of occupational populations, we did not demonstrate an association between different exposure metrics of night shifts and the mean CIMT, a measure of subclinical atherosclerosis. The lack of association may be explained by several reasons. First, CIMT values may be obtained from measurements taken at different carotid segment points on one or both sides, which affected the comparability between different studies. Second, the presence of cardiovascular risk factors in this occupational population may account for the majority of the explained variance in the CIMT and, therefore, the addition of shift work may have a limited effect, which is difficult to detect because of the shared variance with traditional risk factors.

Our results are inconsistent with the association between rotating shift work and subclinical atherosclerosis. Previous studies have revealed that shift workers have significantly higher levels of inflammatory, cardiometabolic risk markers, cardio-ankle vascular index, arterial stiffness and CIMT than daytime workers in adjusted models (15, 22, 27, 28). Analyses of the baseline data of the Brazilian Longitudinal Study of Adult Health (ELSA-Brasil) revealed the increase in exposure to night work was significantly associated with an increase in CIMT among men using a structural equation model (29). However, the lack of association observed in our study has also been reported in the Cardiovascular Risk in Young Finns study (CRYFS): Although shift work was associated with higher mean IMT (β=0.029, P=0.021) and maximum IMT (β=0.029, P=0.028) after adjusting for age only in men, CRYFS found no associations between shift work and mean IMT (β=0.025, P=0.057) and maximum IMT (β=0.026, P=0.057) after further adjusting for all potential risk factors (3). It is noteworthy that these studies often have a coarse assessment of shift work (usually divided into two or more categories, such as day workers and shift workers). However, simply exploring the relationship between coarse exposure indicators of shift work (eg, day versus night worker) and CIMT is not enough to reflect the carotid artery burden and to provide guideline recommendations regarding the risk related to shift schedules, since complete avoidance of rotating night shift work is difficult for socioeconomic realities.

Low socioeconomic status is associated with higher blood pressure, and this association is particularly evident in the level of education (30). Education level might strongly influence adherence through knowledge of hypertension and health behavior, and highly educated people could often improve their working conditions, healthcare and income, which could decrease or delay the occurrence of hypertension (31). In our study, compared with night shift workers, day workers were more educated, received more antihypertensive drugs, smoked and drank less. Night shift workers may have less chance to visit doctors and receive appropriate medication. Regular medication keeps blood pressure in a relatively normal range, and we cannot exclude the influence of regular/irregular medication on blood pressure in the occupational health examination. This partly explains the results that systolic and diastolic blood pressure were significantly higher in night shift workers than day workers, while the prevalence of hypertension was not significantly different.

Several probable pathways are likely to underlie the association between shift work and subclinical athero­sclerosis. One potential mechanism is the presence of psychological and psychosocial stressors (32). Shift workers are subjected to increased stress (such as job strain or community-wide events) than non-shift workers (33). One of the principal mechanisms translating chronic stress into adverse cardiometabolic outcomes is up-regulation of the hypothalamic pituitary adrenal (HPA) axis (34). Chronic elevation of the stress hormone cortisol enhances a set of phenotypic adaptations that promote an overall pro-inflammatory and pro-atherogenic milieu (34). Stress affects the cardiovascular system by stimulating the sympathetic nervous system, impairing endothelial function and creating a hypercoagulable state. All these changes have the potential to result in myocardial infarction or sudden death (35). In addition, shift work may increase the risk of atherosclerosis through adverse effects on sleep (36). Chronically disrupted circadian rhythms, through adipose tissue dysfunction and associated high-risk metabolic traits, create a milieu conducive to atherosclerotic cardiovascular disease (ASCVD) (37).

The major strengths of our study include detailed shift work information and lifestyle information, a large sample size, and accurate calculation of CIMT by ultrasonography. However, our research also has certain limitations. First, we were unable to infer the temporality of shift work and CIMT according to a cross-sectional study. Second, compared with workers who did not take carotid ultrasound, those who did were older, less male, and had higher SBP, DBP, and FBG levels (supplementary table S5). These workers may pay more attention to their physical state due to their age and poor health, introducing volunteer bias. Third, our survey participants were currently participating in the standard four-crew-three-shift system, and different shift systems were only found during the historical period, which made it impossible to directly compare the relationship between different types of night shift systems and the CIMT. Fourth, our study participants are all front-line workers from the production sector, so it was not possible to take into account the occupational category (office or physical workers) that could be confounder of atherosclerosis presence (38). Fifth, those who are competent for long-duration night shift work are more likely to have better physical fitness (the healthy worker effect) or have acclimated to night shift work, which may result in an underestimation of the association between exposure and outcome. Finally, this study was conducted in a steel production occupational setting, and the vast majority of steelworkers are male, which limits the results to the general population.

### Concluding remarks

Different exposure metrics of night shift work were not associated with the CIMT. Further large-scale prospective longitudinal studies are warranted to confirm our findings.
